# Effects of Processed *Polygonum multiflorum* with KIOM Patent on Bone Remodeling-Related Protein Expression in Human Osteoblast-Like SaOS-2 Cells

**DOI:** 10.1155/2020/4168535

**Published:** 2020-04-24

**Authors:** Dae Uk Kim, Jae Yoon Chung, Seong Chul Jin, Mi Hye Kim, Richard Komakech, Ki-Shuk Shim, Yong-Goo Kim, Woong Mo Yang, Youngmin Kang

**Affiliations:** ^1^Oriental Pharmacy, College of Pharmacy, Woosuk University, Wanju-gun, Jeonbuk 55338, Republic of Korea; ^2^School of Korean Medicine, Pusan National University, Beomeo-ri, Mulgeum-eup, Yangsan, Gyeongsangnam-do 626770, Republic of Korea; ^3^College of Korean Medicine, Kyung Hee University, 26 Kyungheedae-ro, Dongdaemun-gu, Seoul 02447, Republic of Korea; ^4^Department of Convergence Korean Medical Science, College of Korean Medicine, Kyung Hee University, 26 Kyungheedae-ro, Dongdaemun-gu, Seoul 02447, Republic of Korea; ^5^Herbal Medicine Resources Research Center, Korea Institute of Oriental Medicine (KIOM), 111 Geonjae-ro, Naju-si, Jeollanam-do 58245, Republic of Korea; ^6^University of Science and Technology (UST), Korean Convergence Medicine Major KIOM, 1672 Yuseongdae-ro, Yuseong-gu, Daejeon 34054, Republic of Korea; ^7^Natural Chemotherapeutics Research Institute (NCRI), Ministry of Health, P.O. Box 4864, Kampala, Uganda; ^8^Herbal Medicine Research Division, Korea Institute of Oriental Medicine (KIOM), 1672 Yuseong-daero, Yuseong-gu, Daejeon 34054, Republic of Korea

## Abstract

This present study evaluated the effects of processed *P. multiflorum* on osteogenesis using Sarcoma osteogenic (SaOS-2) cell lines and osteoclastogenesis of bone marrow-derived macrophage cells (BMM) and to elucidate differences in effect on the expression of bone-related proteins between commercially sold *P. multiflorum* and patented, *in vitro*-propagated Korea Institute of Oriental Medicine (KIOM) *P. multiflorum*. Raw *P. multiflorum* and *P. multiflorum* that were stir-baked and steamed in black bean juice were compared, and western blotting analysis was performed to investigate the expression of bone remodeling-related proteins in SaOS-2 cells. In the cells treated with *P. multiflorum* steamed in black bean juice, the expression of RANKL was decreased, whereas that of osteoprotegerin, alkaline phosphatase, Runx2, and osterix was increased. Owing to these results, we conclude that processed *P. multiflorum* can be used as an alternative treatment for bone diseases such as osteoporosis, osteopenia, periodontitis, and Paget's disease.

## 1. Introduction

In bone metabolism, bones are constantly remodeled by balancing osteoblasts and osteoclasts [1]. In fact, an imbalance between osteoblasts and osteoclasts causes bone loss that can result in various bone diseases such as osteoporosis, osteopenia, periodontitis, and Paget's disease [2, 3]. In recent years, herbal medicines have been used to increase osteoblast differentiation and decrease osteoclast differentiation for treating bone diseases, including osteoporosis [4–6].

The root tuber of *Polygonum multiflorum* Thunb. which belongs to the Polygonaceae family, is a medicinal herb that has been used in Traditional Korean Medicine (TKM) as a blood-tonifying medicine [7]. In Dong-Eue-Bo-Gam, which is the TKM book, *P. multiflorum* is regarded as a medicinal plant with many therapeutic effects, including bone-strengthening [8], potent antiaging, and cognitive-enhancing effects [9], as well as the ability to protect human foreskin melanocytes from oxidative stress and improve pigmentation in hair follicles [10]. *Polygonum multiflorum* also exerts beneficial effects on hippocampal neurons [11].

In TKM, *P. multiflorum* is used after processing because processed *P. multiflorum* exerts better effects than the raw plant [12, 13]. The processing of herbal medicines, one of the core theories in TKM, is aimed at reducing their toxicity and increasing their beneficial effects [14]. To optimize the utilization of *P. multiflorum* in TKM, the Korea Institute of Oriental Medicine (KIOM) developed and patented a standard protocol for rapid in *vitro* production of its seedlings and enlarged root tubers [15]. Therefore, the current study aimed to evaluate the potential effects of *P. multiflorum* produced using the patented protocol Korean Patent submission (no: 10-2019-0120751, September 30, 2019) of KIOM and commercially sold *P. multiflorum* on osteogenesis using osteoblast-like cells Saos-2 and osteoclastogenesis using BMM; this study will serve as a foundation for the use of *P. multiflorum* as a possible treatment of osteoporosis.

## 2. Materials and Methods

### 2.1. Preparation of Commercially Obtained *P. multiflorum*

**Figure 1:**
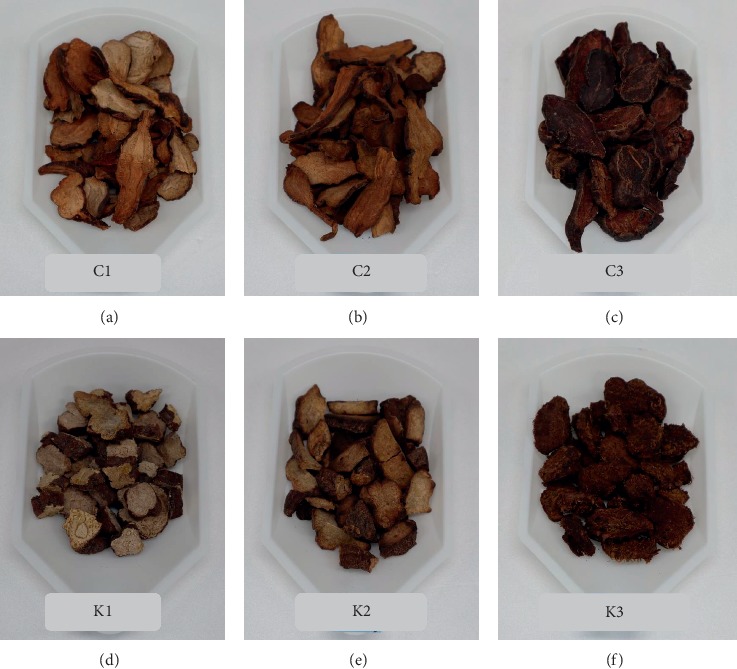
Raw, stir-baked to yellow, and steamed with black bean juice *P. multiflorum*. (C1∼C3: commercial, K1∼K3: KIOM product). (a) commercial raw *P. multiflorum*, C-RPM (C1), (b) commercial stir-baked to yellow *P. multiflorum,* C-SBYRM (C2), (c) commercial steamed with black bean juice *P. multiflorum*, C-SBBJPM (C3), (d) KIOM Raw *P. multiflorum*, K-RPM (K1), (e) KIOM stir-baked to yellow *P. multiflorum*, K-SBYPM (K2), and (f) KIOM steamed with black bean juice *P. multiflorum*, K-SBBJPM (K3).

The 2 kg of the roots of *P. multiflorum* were purchased from Jirisan Hasuo Farming Co. (Sancheong, South Korea) and authenticated by Dr. Kang at Korea Institute of Oriental Medicine (Figure 1(a)) as Commercial Raw *P. multiflorum* (C-RPM), and 1 kg dried roots of raw *P. multiflorum* was baked in a pan with constant stirring at 160°C for 40 min and then maintained in dryer at 45°C for 4 hours (Figure 1(b)) as Commercial Stir-Baked to Yellow *P. multiflorum* (C-SBYPM). The 1 kg of black bean was purchased from Kwangmyongdang Co. (Ulsan, South Korea) and prepared using the method previously suggested [16] in which the obtained beans were boiled in 5 L of water at 100°C for 4 hours to obtain the black beans liquid extract. Then, 4 litres of water was added to the cooked beans and again boiled at 100°C for 3 hours to obtain the second extract. The first and second extracts were then mixed to make the black bean juice. 250 g of *P. multiflorum* and the black bean extract were mixed together in a pot and stirred constantly for 2 hours, then steamed at 60°C for 1 hour, and maintained in an oven at 45°C for 8 hours (Figure 1(c)) as commercial Steamed with Black Bean Juice *P. multiflorum* (C-SBBJPM).

### 2.2. Preparation of KIOM-Obtained *P. multiflorum*

Raw *P. multiflorum* root tubers produced by the Korean patent protocol (patent no. 10-1777833) were provided by Dr. Kang of Korea Institute of Oriental Medicine and were prepared by the same method as in Section 2.1 as KIOM Raw *P. multiflorum (K-RPM)* (Figure 1(d)), KIOM Stir-Baked to Yellow *P. multiflorum* (K-SBYPM) (Figure 1(e)), and as KIOM Steamed with Black Bean Juice *P. multiflorum* (K-SBBJPM) (Figure 1(f)).

### 2.3. Extraction of *P. multiflorum*

The 10 g of each of the six *P. multiflorum* mentioned above was extracted using 100 mL of distilled water for 2 h at 20°C at 200 rpm. After filtration, the obtained extracts were concentrated in a vacuum evaporator and powdered by using a freeze-drying machine for 72 h at −80°C. The dried powder weights of C-RPM, C-SBYPM, C-SBBJPM, K-RPM, K-SBYPM, and K-SBBJPM were 2.56 g, 2.33 g, 1.55 g, 1.37 g, 1.95 g, and 2.37 g (yield: 25.6%, 23.3%, 15.5%, 13.7%, 19.5%, and 23.7%, respectively). The sample was stored at −20°C prior to further studies.

### 2.4. SaOS-2 Cells Culture

Human osteoblast-like SaOS-2 cells, which have similar properties to primary osteoblasts, were obtained from Seoul National University cell bank (Seoul, South Korea). They were derived from an osteosarcoma and cultured in Dulbecco's Modified Eagle Medium (DMEM) supplemented with 10% fetal bovine serum and 1% penicillin at 37°C, 95% humidity, and 5% CO_2_. SaOS-2 cells were seeded at a density of 1 × 10^6^ cells/well in 24-well culture plates. After 24 h of cultivation, the medium was replaced with 3 mL medium supplemented with samples. The sample was diluted with the cell culture medium to obtain concentrations of 100 *μ*g/mL.

BMM were cultured in an *α*-MEM medium having 10% fetal bovine serum and 1% penicillin/streptomycin. BMM were seeded at a density of 1 × 10^4^ cells/well in 96-well culture plates with M-CSF (60 ng/mL). After 2 hour preincubation with each sample (50 *μ*g/mL) on BMM, RANKL (100 ng/mL) were treated for 6 days.

### 2.5. Western Blot Assay

Western blot analysis was performed to investigate the effects of bone metabolism in SaOS-2 cells, osteoclast differentiation factor; RANKL and OPG, osteoblastogenesis factors; alkaline phosphatase (ALP), Runt-related transcription factor 2 (Runx2), and Osterix expressions. Each sample of SaOS-2 cells was vortexed in RIPA buffer (Thermo scientific, Rockford, USA). 20 *μ*g cell protein from SaOS-2 was denatured with 5% SDS buffer. The prepared protein samples were loaded on 10% polyacrylamide gels, separated by electrophoresis, and then electrotransferred to activated polyvinylidene fluoride (PVDF) membranes. Membranes were blocked by 3% bovine serum albumin (BSA) in tris-buffered saline (TBS) containing 1% Tween 20 (TBS-T) and incubated with the specific antibodies at 4°C for 12 h (*β*-actin, OPG, RANKL, Osterix, ALP, RUNX2; Santa Cruz Biotechnology, Inc., CA), 1 : 1000 dilutions in TBS-T). After washing of the membranes for 10 min 3 times, membranes were incubated with anti-rabbit and anti-mouse alkaline phosphatase-conjugated secondary antibody (1 : 2000 dilution in TBS-T) for 1 h at 24°C. The membranes were washed three times in TBS-T for 3 min and then reacted with HRP-polymerized secondary antibody for 60 minutes, after completion of the reaction, using a chemiluminescent detection system (Phototope®-HRP western blot assay Detection kit, New England Biolab).

### 2.6. MTT Assay

The cell viability was assessed using the 3-(4,5-dimethylthiazol-2-yl)-2,5-diphenyl tetrazolium bromide (MTT) solution. For a short time, 1 × 10^5^ cells/well were seeded in 96-well plates and allowed to adhere for 12 h. The cells were treated with 100 *μ*L of 100 *μ*g/mL samples each for 24 hours, and MTT (2 mg/mL) was added 50 *μ*L/well and incubated for 2 h, followed by solubilization of the formazan crystals by adding dimethyl sulfoxide (Sigma-Aldrich, Seoul, South Korea) 50 *μ*L/well for 30 min. The color developed was read as optical density using a Gene5 (Biotek, Seoul, South Korea) at 570 nm.

### 2.7. Statistical Analysis

All data were expressed as a means ± standard error of the mean. One-way ANOVA followed by the Tukey test (compare all pairs of columns) was used for the statistical analysis. In all analyses, *p* < 0.05 was taken to indicate statistical significance.

## 3. Results

### 3.1. Effects of samples on osteoclast differentiation mediators, RANKL, and OPG in SaOS-2 cells

**Figure 2:**
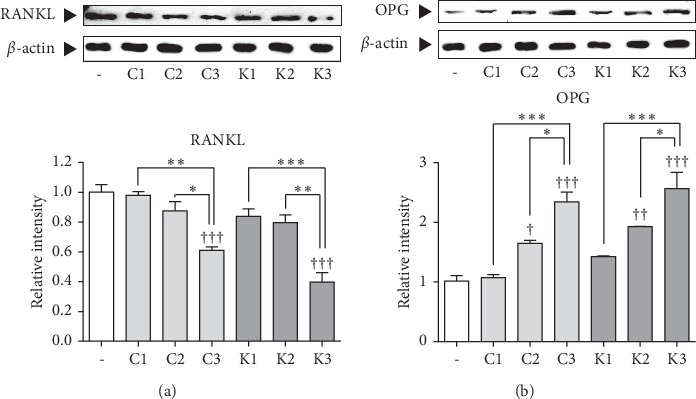
Effects of samples on osteoclast differentiation mediators, RANKL, and OPG in SaOS-2 cells. (a). Effects of samples on RANKL protein levels in SaOS-2 osteosarcoma and expression of RANKL, osteoclast differentiation factor, in SaOS-2 osteosarcoma cells. (b). Effects of samples on OPG protein levels in SaOS-2 osteosarcoma. Expression of OPG, RANKL inhibitor, in SaOS-2 osteosarcoma cells ^†^*p* < 0.05, ^††^*p* < 0.01 and ^†††^*p* < 0.001 vs. nontreated cells. ^*∗*^*p* < 0.05 and ^*∗∗∗*^*p* < 0.001.

C-RPM, K-RPM, C-SBYPM, and K-SBYPM were not effective on the decrease of the RANKL expression. On the other hand, C-SBBJPM and K-SBBJSPM treated with black bean juice decreased the expression level of RANKL by 62.3% and 60.2%, respectively, compared to the control group. C-SBBJPM significantly decreased RANKL expression compared to C-RPM and C-SBYPM. K-SBBJPM significantly decreased RANKL expression compared to K-RPM and K-SBYPM (Figure 2(a)).

C-RPM and K-RPM were not effective on the increase of OPG expression in SaOS-2 cells. On the other hand, C-SBYPM and K-SBYPM and C-SBBJPM and K-SBBJPM increased the level of OPG by 62.7%, 90.6%, 131.2%, and 153.1%, respectively, compared to the control group. C-SBYPM and C-SBBJPM increased OPG expression compared to C-RPM. C-SBBJPM increased the OPG expression compared to C-SBYPM. Likewise, K-SBYPM and K-SBBJPM increased the OPG expression compared to K-RPM. K-SBBJPM increased the OPG expression in SaOS-2 cells compared to K-SBYPM (Figure 2(b)).

### 3.2. Effects of Samples on Osteoblast Differentiation Mediators, ALP, Runx2, and Osterix in SaOS-2 Cells

**Figure 3:**
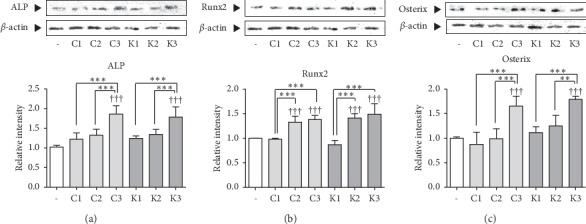
Effects of samples on osteoblast differentiation mediators, ALP, Runx2, and Osterix in SaOS-2 cells. (a) Effects of samples on ALP protein levels in SaOS-2 osteosarcoma. Expression of ALP, osteoblast differentiation factor, in SaOS-2 osteosarcoma cells. (b) Effects of samples on Runx2 protein levels in SaOS-2 osteosarcoma. Expression of Runx2, osteoblast differentiation factor, in SaOS-2 osteosarcoma cells. (c). Effects of samples on Osterix protein levels in SaOS-2 osteosarcoma. Expression of Osterix, osteoblast differentiation factor, in SaOS-2 osteosarcoma cells. ^†^*p* < 0.05 and ^†††^*p* < 0.001 vs. nontreated cells. ^*∗∗*^*p* < 0.01 and ^*∗∗∗*^*p* < 0.001.

C-RPM and K-RPM and C-SBYPM and K-SBYPM were not effective on the increase of ALP expression in SaOS-2 cells. On the other hand, C-SBBJPM and K-SBBJPM increased the level of ALP by 81.9% and 74.6%, respectively, compared to the control group. C-SBBJPM significantly increased the ALP expression compared to C-RPM and C-SBYPM. K-SBBJPM significantly increased the ALP expression compared to K-RPM and K-SBYPM (Figure 3(a)).

C-RPM and K-RPM did not change the expression of Runx2 in SaOS-2 cells. On the other hand, C-SBYPM and K-SBYPM and C-SBBJPM and K-SBBJPM increased the Runx2 expressions by 32.8%, 38.3%, 41.3%, and 49.0%, respectively, compared to the control group. C-SBYPM and C-SBBJPM increased the Runx2 expression compared to C-RPM. K-SBYPM and K-SBBJPM increased the Runx2 expression compared to K-RPM (Figure 3(b)).

C-RPM and K-RPM and C-SBYPM and K-SBYPM did not increase the expression of Osterix. On the other hand, C-SBBJPM and K-SBBJPM increased the expression of Osterix by 65.5% and 79.3%, respectively, compared to the control group. C-SBBJPM significantly increased the Osterix expression compared to C-RPM and C-SBYPM. K-SBBJPM significantly increased the Osterix expression compared to K-RPM and K-SBYPM (Figure 3(c)).

### 3.3. Difference between Commercial *P. multiflorum* Samples and KIOM Samples

There was no difference of RANKL and OPG expression between commercial *P. multiflorum* samples and KIOM samples. In addition, there was no difference of ALP, Runx2, and osterix expression between commercial *P. multiflorum* samples and KIOM samples.

### 3.4. Effect of *P. multiflorum* on SaOS-2 Cells Toxicity

**Figure 4:**
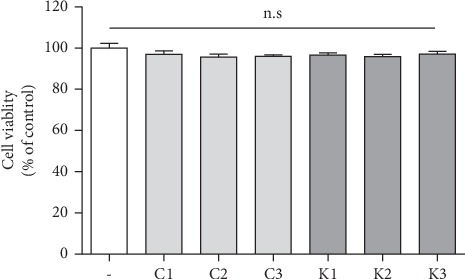
Cell viability of SaOS-2 osteosarcoma cells. Cell viability of SaOS-2 osteosarcoma cells. n.s-no significance.

The effect of *P. multiflorum* on toxicity in SaOS-2 cells was examined by the MTT assay. The cells were treated with different doses of *P. multiflorum*, and no toxicity was found (Figure 4).

## 4. Discussion

The balance between osteoblasts and osteoclasts is one of the key components of bone metabolism [17]. Bone homeostasis cannot be maintained if there is excessive osteoclastic bone resorption or insufficient osteoblastic bone formation [18]. Attenuation of RANKL or activation of OPG (RANKL inhibitor) might be helpful to inhibit the differentiation of osteoclasts, resulting in alleviation of bone resorption [2]. In this study, the expression of RANKL was significantly decreased by C-SBBJPM and K-SBBJPM treatment, while C-RPM, C-SBYPM and K-RPM, K-SBYPM did not change the expression of RANKL in SaOS-2 cells. This result showed that steamed black bean juice of *P. multiflorum* apparently affected the production of RANKL in osteoblasts. In terms of OPG, all processed *P. multiflorum* samples including C-SBYPM, C-SBBJPM, K-SBYPM, and K-SBBJPM except C-RPM and K-RPM were effective on the expression of OPG in SaOS-2 cells. Nevertheless, steamed black bean juice of *P. multiflorum* was more effective than that of stir-baked to yellow *P. multiflorum* in both of commercial *P. multiflorum* samples and KIOM samples. Steamed black bean juice of *P. multiflorum* increases the inhibitory effects of *P. multiflorum* on bone resorption by osteoblasts by decreasing RANKL expression and increasing OPG expression.

At the stage of osteoblast differentiation, ALP, Runx2 and Osterix, and osteoblast-specific transcription factors are released from osteoblasts, leading to induction of bone formation [2]. This experiment showed that the levels of ALP and Osterix were significantly increased in C-SBBJPM and K-SBBJPM-treated cells. In part of Runx2 expression, C-SBYPM, C-SBBJPM, K-SBYPM, and K-SBBJPM increased the Runx2 compared to raw C-RPM and K-RPM. However, the level of Runx2 in cells treated with C-SBBJPM and K-SBBJPM was higher than C-SBYPM and K-SBYPM. The steamed with black bean juice method apparently upregulated the effects of *P. multiflorum* on osteoblast activation rather than the stir-baking method which is consistent with the result from osteoclast-related factors.

The cultivation of *P. multiflorum* typically takes long time to propagate which leads to decreasing the yield and quality [19]. However, with the KIOM-patented root tuber enlargement protocol for *P. multiflorum*, rapid in vitro propagation can be carried out without compromising its chemical composition [15]. This experiment shows that the overall tendency of expressions of bone remodeling-related factors between commercial *P. multiflorum* samples and KIOM patented samples is very similar. To clarify the pharmacological potential of K-RPM and K-SBBJPM against bone disease, we are currently investigating the *in vivo* efficacy of the samples in preclinical animal models.

## 5. Conclusion

Processed *P. multiflorum* regulated osteoclast differentiation mediators, RANKL, and OPG and increased osteoblast differentiation mediators, ALP, Runx2, and osterix. In addition, there was no significant difference in expressions of RANKL, OPG, ALP, Runx2, and osterix between commercial *P. multiflorum* samples and KIOM samples. However, although this study has showed promising evidence that processed *P. multiflorum* might be considered as a possible treatment for bone diseases, there is need for further preclinical and clinical studies to enhance future drug development from it for the treatment of bone diseases. Additionally, future study should also consider comparing the phytochemical compositions in the different *P. multiflorum* roots.

## Data Availability

The data for this current study are available from the corresponding author upon reasonable request.
